# Clinical Record of a Group of Surgical Cases of Special Interest Treated during the past Year—Hospital, Dispensary and Private Cases

**Published:** 1898-09

**Authors:** Thomas H. Manley

**Affiliations:** New York


### Southern Medical Record.

A MONTHLY JOURNAL OF MEDICINE AND SURGERY.

VOL. XXVIII. ATLANTA, GA., SEPTEMBER, 1898. NO. 9.

### Original Articles.

### CLINICAL RECORD OF A GROUP OF SURGICAL CASES OF SPECIAL INTEREST TREATED DURING THE PAST YEAR—HOSPITAL, DISPENSARY AND PRIVATE CASES.

By THOMAS H. MANLEY, M. D., New York.

While the sciences must necessarily constitute the groundwork of progressive medicine, anatomy, physiology, chemistry, pathology and bacteriology each occupying an important sphere, yet the new beginner who starts out on his career with no other guide than a crammed, ill-digested knowledge of these branches is doomed to early chagrin and disappointment. He has not yet learned the practical side of medicine, and until he does he is little more than a dangerous experimenter, bound to inflict much harm before he falls into the right path.

A glaring illustration of the wide difference between theories and practice we have recently beheld in our late war with Spain.

The credulous, unsophisticated amateur would assume from the many over-colored vaporings of enthusiasts that by the utilization of the latest acquisitions of science and art military medicine and surgery must be revolutionized and rewritten, our advances and improvment having been so great. But what are the facts?

Well, let us candidly confess the truth and admit that though thirty-three years have passed since the Civil War, we have no evidence that in those three decades any great advances have been made in medical or surgical prophylaxis of campaign life. Antiseptics have sustained a bad setback, the mortality from abdominal section has been great, while those penetrating wounds of the peritoneum treated by the let-alone plan have generally done well.

Dr. Senn's late contribution from the Relief points to practically nothing new, and again comes the worst blow to sanitary science, our boasted mastery over the contagium-vivum is little more than a myth. Our panic-stricken commanders at the front pitifully appeal to the government to transport their armies north before the pestiferous diseases of Cuba blot them out, as neither hygiene nor medical art is competent to prevent or mitigate them. This short war has taught us, as the Madagascar campaign did the Italians, a practical lesson, and led us to question many things which our laboratory authorities would have had us believe were as fixed and immutable as the fabled laws of the Medes and Persians.

The practical, or the clinical, side of the healing art is its very soul and life. Let the novice witness the treatment of a half dozen fracture cases, and it will do him more good than a month's reading on theory and speculation. It is therefore of vital importance that our collateral studies of the coguate branches of medicine be illumined, leavened aud strengthened by clinical studies and immediate personal observation.

The record of cases by any unbiased observer is always of interest and value; therefore in this instance, with the very limited space at my command, there will be noted a few cases of a cl iss of lesions of more than passing interest.

### I. TUMORS OF THE LYMPHOID ABSORBENTS.

Briefly and roughly speaking, though tending to suppuration or extensive and rapid hypertrophy, these tumors are either specific in the inguinal region or tuberculous in the cervical.

Thirty-four such cases have come under my care in the past year, most of them having been sent to me for surgical treatment. This meant that palliative measures had entirely failed.

My course in these cases is incision and ablation complete.

In all these cases a free, wide incision provides us with great security in dissection, but in the submaxillary, or supra-clavicular areas of the female, for cosmetic reasons we are often forced to make as small an incision as possible, with a view of leaving a minimum cicatrix.

Strumous neoplasms in the internal cervical areas are removed with ease and rapidity in most instances by an experienced operator. If the inexperienced undertakes these operations on the neck, he should first make a few thorough dissections on the cavader; else serious consequences through damage of some of the many vital structures which pass down over the cervical isthmus is possible. After complete enucleation here, we should close with fine silk rather than gut, which leaves a rosary of scars.

The excision of bubonocele is not difficult, because we may here widely expose the deep parts by a free incision; caution only is required that in dissecting we nibble carefully with the blade when we sink down on the great blood trunks and always keep close to the capsule. It is curious to note that in venereal bubo, do what we will, primary union of the incision seldom occurs. It has been said that this is because the integument can not be well disinfected here. This is an error evidently, for the reason when we cut in this situation over the inguinal fold for hernia, spermato :ele or other condition, the parts promptly close in.

II. FRACTURES.

During the past twelve months fifty-three cases of fracture have come under my care and notice.

It is a most singular thing, but the fact remains that of late years there has been neither in England nor America a real up-to-date work on the management of complex fractures. Here the young man is quite at sea, for one author will tell him that immobilization is necessary from one to three months. He tries it on a fat old woman with an intracapsular fracture of the femur, and he is rewarded with a vast bed sore, and later, possibly, death. He next tries it a Colles. After a month when the fixation is removed, he finds every joint carpal and digital anchylosed. Turn again to a French surgeon's late work on fracture-treatment and we will find that he urges the ambulant treatment with no splints at all, and motion with massage from the very first day.

The fact is that the proper treatment of fractures in general is a science in itself, of which the profession as a whole know very little. But few are familiar with Ollier's monumental work on this topic, or the fact that a fracture, like a lesion through the soft parts, under proper treatment may, often do, solidly unite by primary union. My course in fracture treatment is based on simple fundamental principles, the most cardinal being not to in any manner embarrass the circulation.

Our greatest and most substantial advances in this direction have been through utilizing osteoplasty in compound fracture and the application of prothetic apparatuses. An enormous gain, too, has been possible by embalming all serious shattered fractures until the demarkating line is formed, if it form at all; in other words, dispensing *in toto* with primary amputations. It has been my invariable custom in all fractures, simple and compound, not to apply any permanent adjustment until all inflammatory reaction has subsided.

The Roentgen rays have been an aid in some cases, but there are many non-displaced fractures about the joints where their revelations are most delusive or negative. As a diagnostic aid in fractures ts value has been rather overestimated.

One of the most serious cases of fracture of a large bone shaft that has come under my charge this year was sent to me by my friend, Dr. R. H. Cowan, the chief surgeon of the Norfolk & Western Railroad. The young man thirteen months before had sustained an extensive compound comminuted fracture of the right femur, with several other serious injuries of the body.

He finally made a good recovery, but at the side of fracture there remained a cushion joint, with a sharp, angular deformity, rendering the limb both painful and useless for support. It was simply a question between amputation and restoration of position and function by some osteoplastic operation.

He was a young man with a fine constitution, and hence there was no doubt about the presence of ample regenerative action in the tissues. But the femur is the one bone shaft which is exceedingly intolerant to mechanical manipulation after a recent fracture, although for pathologic states we may accomplish much through surgery. Briefly, it may be said that an operation was performed, an exceedingly difficult and bloody one, by which all the diseased bone was removed and the ends of the fragments solidly wired. The case has followed an uneventful course, promising to yield the happiest resu.ts as to position, strength and function.

III. HYDROCELE VARIX, NEOPLASM, INGUINAL VARIX, SEROUS CYSTS, HERNIO-SEROUS CYSTS OF THE SCROTUM.

Thirty-seven cases of the above class are recorded in the annual of the West Side German Dispensary, and in my service during the same period as preceding cases, with what have been seen in my private practice and consultation in the same time, would nearly double the number.

To classify and arrange this group on an anatomical or pathological basis in the present instance, important and valuable as it would be, is impossible. Attention is particularly called to them here, because of their comparative frequency, the difficulty in their diagnosis often, and the value of an aseptic incision under many circumstances, both to clarify diagnosis and simultaneously as a therapeutic resort institute surgical treatment. In four old chronic cases, hernia, hydrocele and varix coexisted. My rule of practice has been with this class to advise and practice radical surgery in the young of vigorous health, but to discourage operating in the feeble or aged, unless threatening symptoms are manifest.

In infants and youths, when diagnosis is at all doubtful, a free incision is invariably practiced.

When this is done aseptically and the patient keeps the bed for a week or two recovery promptly follows with few exceptions.

VAST HERNIO-CYSTIC TUMOR OF SCROTUM.*

Among this group was one remarkable case of right inguinal hernia, with a vast multilocular cyst of the scrotum. The entire mass was supported by braces crossing both shoulders and reaching down below the knees. I had it weighed in an empty starch box turned up on its end. Weight, sixteen pounds and seven ounces. Diameter over its antero posterior, or long axis, twenty-seven inches; circumference, twenty-six inches. Contained a fluid of oily consistence and coffee color. This mass was treated by free evacuation, more than a gallon of fluid having been evacuated. On operation it was found to consist of two separate dermoid cysts, one having a thick, cartilaginous investment; both contained an abundance of putty-like mass and gave issue nearly two gallons of chocolate-colored fluid.

After operation the hernia was readily reduced.

IV. HEMORRHOIDS, ITCH, ULCER, PROLAPSE OR STRICTURE OF THE ANO-RECTAL OUTLET.

*Cases.*—In period stated, thirty-seven cases of rectal affections have come under my notice and treatment.

The most common and troublesome affection of the rectal outlet is piles, and, as there are multiple varieties of them, it goes without saying that treatment must be modified in various circumstances, though one common rule should be observed under all circumstances, viz., not to needlessly multilate or cut when tentative measures may succeed.

It has therefore been my custom in practically all but ulcerating piles to be content with cocaine analgesia, dilatation and pressure massage. No scalpel or instrument can reach the source of hemorrhoidal disease in the portal system, or the vasomotor plexus.

My procedure, when feasible, dispenses with the dangers of pulmonary anesthetics, large hemorrhage or infection after operation; besides its effects are most gratifying and effective.

Rectal ulcer is frequently the cause of anal pruritus. Ulcers dependent on tubercle syphilis or cancer precede stricture. When stricture sets in hemorrhage and fecal leakage quite generally attend it. In women we must be cautious not to confound a retroverted fundus uteri on the rectum for a new growth. Prolapse of the anus in the young is usually self-curable, but one case of a most aggravated description came

*This remarkable and unique case will be reported in detail after cure is complete.

under my care in the early spring, associated with extroversion of the bladder, the patient being but four months old.

In middle life and later no rectal lesion will call for greater tact, skill and judgment in its treatment.

This condition possesses quite all the etiological features of a hernia, and hence similar principles apply to the treatment of both.

In five cases seen by me among the enumerated group three were in females. In this sex we find it the most common and quite invariably associated with a collapse of the bladder, vagina or uterus.

### V. COPROSTASIS COLO-RECTAL INERTIA.

I can find no mention of this pathological condition in the literature of medicine. Much will be found on constipation, but this is something more. The colon and rectum are jammed, packed full, with a super-added diarrhea and fecal leakage.

During the past winter after an operation on a gentleman for phagadenic appendicitis he complained to me of a "drip" coming away from his bowels, which had annoyed him for some weeks before the operation.

On examination the rectum was found packed with hard fecal masses. The anal sphincter had to be widely dilated and large irrigations employed before it was possible to dislodge the enormous accumulation. Under similar circumstances of general enfeeblement of the body, and when the patient complains of a "soiling of the clothes," it is always well to explore and empty the rectum at once.

In the case cited, immediately on mechanical evacuations, pain, distension and languor quickly disappeared, appetite, sleep and strength were restored.

### VI. CASES OF TRAUMATIC AND PATHOLOGICAL CONDITIONS OF THE EXTREMITIES.

A brief notice only here can be given to certain inflammatory conditions of the joints, traumatic or pathological.

More than one hundred cases of this class have been handled at my clinic in dispensary, in hospital and private practice within one year. A little more than a third were traumatic, about a quarter either tubercular or syphilitic; there were seven gonorrheal, the remainder were of a neuralgic or rheumatic character.

The unsatisfactory results in the treatment of this class of cases often arises from two causes. The first is an imperfect knowledge of the anatomy of the extremities, and the next is through a hasty or imperfect investigation into the causation. For example, how often one is treated for synovitis when the effusion is inflammatory and entirely external to the joint in a neighboring bursa? And how often may a gonorrheal or tubercular arthritis be confounded with rheumatism? Again, it is now well known that articular degeneration may have its source in the spinal centres.

Late years have witnessed a marked back-setting in those extensive dissections of joints so recently practiced on the articulations.

Arthrectomy in my practice has been proved to be very rarely required. Young children outgrow many joint lesions. In adults tubercular arthritis very often coexists with pulmonary implication.

A large proportion of the cases coming under my care were entered for the relief of pain and stiffness in the joint. In many the dominant surface features pointed to rheumatism, though the history indicated trauma. This is an important point to note, for my experience has forced me to believe that in many of a gouty or rheumatic diathesis a trivial injury of an articulation may stir into activity a rheumatic, chronic inflammation. It therefore follows that in many of these constitutional treatment must go hand in hand with local measures.

For local treatment in the majority of cases of joint pain nothing is so prompt and decisive in its effect as acupuncture, followed by accelerating the joint action and bandage support. As a liniment nothing excels for these cases the following, first employed in this country by myself. It sometimes seems to almost work wonders:

| | |
|---|---|
| ℞—Acidi salicylici | dr. iv ss |
| Tr. opii | dr. iii |
| Spts. vini rect. dil | oz. ii |
| Ol. terebinthin | oz. i |
| Ol. dulcis, qs. ad | oz. viii |
| M. Sig.—Liniment. | |

We must eliminate the opium tincture in growing children. It may be applied by inunction or by saturated flannels. The hand must be well oiled which is to rub it in.

### VII. HERNIAL CASES.

Fifty-seven cases of various types of hernia came under my care in period stated.

Thirty were in infants and children, twelve in middle-aged and young adults, and fifteen in old people. It is curious to note how common this infirmity is in men over sixty, especially with those who suffer from prostatic or vesical disease, and it seems remarkable, too, that their ruptures tend to take on the infantile type, *i. e.*, they are prone to cystic complications.

Surgery can do much for various types of hernia, though it can not cure or relieve all.

My rule has been, to recommend the truss when it can be worn with comfort, when the rupture shows no signs of complications, is not augmenting in volume nor threatening strangulation. This rule is deviated from, however, with young women, in whom kelotomies are generally followed by the most gratifying results, and besides in those with whom the males blemish may constitute an impediment in physical examinations. As operations, *secundem artem* for non-strangulated hernia is quite devoid of danger, we rarely decline to perform them on healthy subjects in early life.

### VIII. POST-PARTURIENT EVENTRATION.

Two cases of remarkable collapse forward of the abdominal walls after labor came under my care recently, both seeking relief from the so-called "pot belly" by surgery. Both were young women, who had gone through normal labors, but below the umbilicus it was quite evident that over the anterior lateral aspects of the abdomen no vestige of muscular tissue remained. The floating viscera tumbled in a thin, attenuated pouch over the pelvic brim, and with the hand one could readily pick up the parchment-like envelope and readily feel the vermicular movement of the intestine.

Both women were in good health and showed no signs of disease of any of the pelvic organs.

I had once seen, post-mortem, a similar condition in a woman who had for thirty years a large uterine fibroid.

After a careful study of these cases I could not see my way clear to conscientiously recommend any description of surgical operation, although before they came to me both had been assured that they could be "cured by a very simple operation."

### IX. SURGERY OF THE CAVITIES.

*Cases.*—Surgery of the cavities has been vastly overdone, or, rather, indiscriminately employed. The scalpel can do little for pulmonary lesions except in suppurative conditions; but every year we are steadly enlarging the field of abdominal and pelvic surgery. Four cases of large fibroid, fifteen of salpingitis and eight of uterine cancer are enumerated in my annual collection. There were nine cases of pyelonephrosis, calculous and tuberculous; twenty-seven cases of appendicitis, one of renal carcoma, six of pyloric obstruction, all malignant; one of cancerous stenosis of the esophagus, and one of ileus.

No branch of surgery offers better and more satisfactory results in properly selected cases, and when dealt with by skilled, experienced hands, than that applied to the abdomen and pelvis; while, on the contrary, indiscriminate, rash or unskillful operating here is murderous. The master of this work must know practical anatomy well, have done plenty of vivisection and have had clinical experience. It has been my experience that when we operate here in the absence of acute inflammation, results are very generally satisfactory.

Experience has taught me, too, that safe and rapid operating is greatly enhanced by a free incision, which when properly closed in no wise favors hernia any more than a small one.

From what I saw last year in the English and French hospitals I have been led to largely discard drainage in all abdominal cases unless when dealing with a suppurative condition, or secondary hemorrhage is feared.

### X. TUMORS OF THE MAMMARY GLAND.

Since October 1, 1897, sixteen of these cases have come under my notice or care, four in consultation. Of this number four were of a benign character.

Although the general rule now is to clear away the whole chest wall in the ablation of cancerous breasts, still my own views on this lesion and the results which I have seen after those large dissections have not inclined me not to adopt it. I have been led to the more restricted operation because no *single case* of clearly established cancer in any situation has ever been seen by me cured by any operation, and further, when the axillary hollow and the supra-clavicular spaces are entirely cleared of obstruction the venous and lymph currents is certain to follow, with more or less neuralgia in the upper extremity, edema and marked elephantiasis. Operation for true cancer, then, should always be regarded as little more than a palliative resort.

Two of the cases in this number had been pronounced cancer, one of which was a dermoid cyst and the other a deep-seated tubercular abscess. No doubt, however, early extirpation offers the greatest security against early return, and should be particularly recommended in young people; but with decrepit, old women mammary cancer is generally quite painless and pursues a very chronic course, hence in them sanguinous measures should not be urged.

### XI. STRICTURES IN THE CERVICAL SEGMENTS OF THE ESOPHAGUS.

Stricture of the esophagus is very often one of the first symptoms of cancer in the posterior anediastinum, though we will rarely meet with it until after middle life.

My patient was a young lady who came to me in May. Ten years previously I had removed her right hip joint for tubercular disease.

But now she was hale and hearty and was enjoying good health until about ten months before she reported, when she had difficulty in swallowing, beginning by requiring a special effort to swallow solids. Later this became more pronounced, when no solid food whatever could be forced down without a prompt regurgitation. For more than a month she subsisted entirely on liquids.

It was at first thought that the trouble was neurotic, but antispasmodic remedies produced no influence, and besides the clinical history was against it. She now had a large tuberculous ulcer of the leg. She was twenty-two years old and unmarried. Could she have had a tuberculous ulcer of the esophagus, which on healing left a contracted state of the lumen, or might it not possibly have been syphilitic? I ca[illegible] find no case on record of tubercular stenosis of the esophagus, and her life has been such as would lead me to reject venereal, absolutely.

The surface examination of the neck revealed nothing. On passing in a small bougie, an obstruction was encountered on a level with the sixth cervical vertebra. It would not permit a larger one to pass. I then took the smallest size and easily passed it on towards the stomach. This was repeated when larger and larger sizes were passed, until one the full diameter of the esophagus went through. The results were most gratifying, and now she has no trouble in deglutition, swallowing with ease any description of food coming before her.

### XII. TRAUMATISMS INVOLVING THE MUTILATION OF THE JOINTS AND SOFT PARTS.

*Cases.*—A large number of injuries involving fleshy parts have been seen during the past year, most of them being lacerated or fractured, and attended by contusions.

It strikes one as rather remarkable that young practitioners will persist in sewing up lacerated wounds. Imbued with a profound conviction that provided only a wound is amply scrubbed and pickled in an antiseptic solution, immutable and fundamental laws of surgery may be ignored with impunity.

An example of this came under my notice very recently.

A high official of this city, rusticating with his family in the Catskill Mountains, one morning mounted his horse for a ride. The animal shied at an object and became unmanageable, when the rider was thrown off, sustaining a deep, lacerated wound over the metocarpo-phalangeal joint of the little and ring fingers, right hand. The gash bled very freely. He was promptly attended by a physician, who immediately sealed the opening with deep suture. Three days later, as serious symptoms were developing, he was brought home. The following day I was invited by his New York physician to see the wound. Things now were indeed in a menacing state. His hand was bloated up, the sutures had ulcerated out, a purulent tendovaginitis had extended into the deeper part of the hand and the first phalanx of the little finger was bare on its inner aspect. Along with this he had a temperature of 104, with recurring rigors and bodily weakness. Incisions, counter incisions, irrigation and drainage were employed, while he was plied freely internally with whisky and quinine. He had a narrow escape from losing the hand, but the little finger had to be amputated.

In some of these cases of lacerations of the hand, in the summer season, unless prompt and efficient remedies are supplied, gangrene may set in with destructive energy and destroy with appalling rapidity.

Some years ago I saw after a deep laceration of the palm by a butcher's hook, in which the wound was sewed up within thirty-six hours, mortification had set in so rapidly and extensively that I had to amputate at the humero-scapular articulation at the shoulder to save life.

The most valuable of all remedies for all deep lacerations are cold alcoholic solutions, but always leave the wound widely open.

Another mistake in treating lesions which open into the joints or involve a fracture of bone is the employment of strong antiseptic solutions, which by their irritating properties often start up a low grade of endosteal or perichrondrial inflammation, difficult to subdue without resort to resection or amputation.

### RESUME AND SPECULATIONS.

Let no one delude himself into supposing that any advances, no matter how great, during the past twenty-five or thirty years have in any manner revolutionized the fundamental principles of surgery.

A greater latitude of action is now possible, through anesthetics, by which our patient is rendered temporarily dead to all sensations, and our work is not entirely unlike an autopsy. This permits leisure, accuracy and precision, without which progressive operative surgery would be impossible.

Lister taught the world the doctrine of cleanliness; the inventions, industries and discoveries of modern times have contributed vastly to progressive and special surgery, and above them all, the masses have torn loose from the chains of the despot, the democratic spirit has taken deep root, and poor, struggling humanity is now permitted to enjoy some of the fruits of toil and labor; every one is better fed, domiciled and clothed, and the accursed "famine fever" of former times is no longer possible. Therefore why the patient stands the shock of operation with comparative immunity and the death rate has been enormously lessened.

